# Perioperative management of a patient with a giant thyroglossal duct cyst: a case report

**DOI:** 10.1093/jscr/rjac309

**Published:** 2022-06-30

**Authors:** Yoh-ichiro Iwasa, Kentaro Hori, Ken Hiramatsu, Yoh Yokota, Tomohiro Kitano, Ryosuke Kitoh, Yutaka Takumi

**Affiliations:** Department of Otorhinolaryngology-Head and Neck Surgery, Shinshu University School of Medicine, Matsumoto City, Japan; Department of Otorhinolaryngology-Head and Neck Surgery, Shinshu University School of Medicine, Matsumoto City, Japan; Department of Otorhinolaryngology-Head and Neck Surgery, Shinshu University School of Medicine, Matsumoto City, Japan; Department of Otorhinolaryngology-Head and Neck Surgery, Shinshu University School of Medicine, Matsumoto City, Japan; Department of Otorhinolaryngology-Head and Neck Surgery, Shinshu University School of Medicine, Matsumoto City, Japan; Department of Otorhinolaryngology-Head and Neck Surgery, Shinshu University School of Medicine, Matsumoto City, Japan; Department of Otorhinolaryngology-Head and Neck Surgery, Shinshu University School of Medicine, Matsumoto City, Japan

## Abstract

Thyroglossal duct cysts (TGDC) are the most common type of congenital neck masses, which generally present in young adults. We present a rare case of a giant TGDC in a 77-year-old patient who required atypical perioperative management. The patient presented with a large soft mass on his anterior neck. Computed tomography showed a lobulated cystic mass measuring 18 × 16 cm, extending from the tongue base to the inferior level of the clavicle. Because difficult intubation was expected, the cyst was punctured and most of the fluid was drained prior to surgery. The swelling of the tongue base was remarkably reduced, and intubation was performed safely. The cyst was extracted using the Sistrunk procedure and tracheotomy was performed. Histopathological examination confirmed the diagnosis of TGDC. Preoperative volume reduction of the cyst and tracheotomy should be considered for oral intubation and postoperative airway management, respectively, in patients with large TGDC.

## INTRODUCTION

Thyroglossal duct cysts (TGDC) are the most common type of congenital neck mass, arising from epithelial remnants of the thyroglossal duct [1]. They usually present as painless anterior neck masses within the first two decades of life and are rare in older adults [2]. TGDC can occur anywhere along the thyroglossal duct of the developing thyroid gland, from the foramen cecum of the tongue to the suprasternal fissure, and are commonly located in the infrahyoid (70.2–75.9%), followed by the suprahyoid [3, 4]. Curative treatment consists of surgical resection of the cyst along with the middle section of the hyoid bone; this is known as the Sistrunk procedure [5]. Here, we present a rare case of an older adult with a giant TGDC that required unusual perioperative management.

## CASE REPORT

The patient was a 77-year-old man who first presented at a clinic in 2012 with anterior neck swelling. Computed tomography (CT) showed a 3.5 × 3.4-cm infrahyoid cystic mass, and fine-needle aspiration cytology suggested TGDC. He refused surgery and did not visit the clinic for the subsequent 9 years.

**Figure 1:**
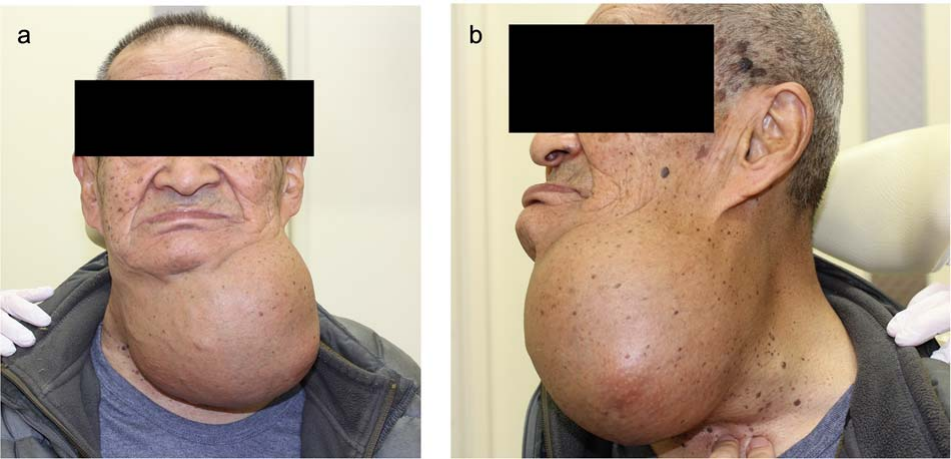
Preoperative photograph (**a**) frontal view (**b**) lateral view.

**Figure 2:**
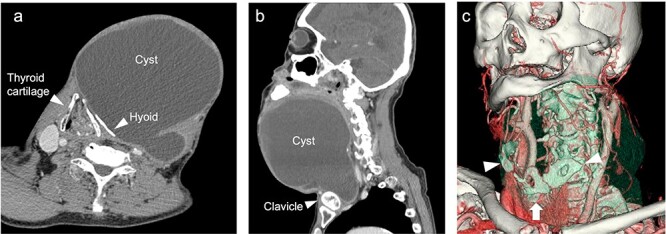
CT scan imaging of the cyst (**a**, **b**). The thyroid cartilage pressed toward the right side and dislocation of the hyoid bone (**c**) 3D-imaging of CT scan. Deformed hyoid bone (arrowhead) and pressed thyroid cartilage (arrow).

**Figure 3:**
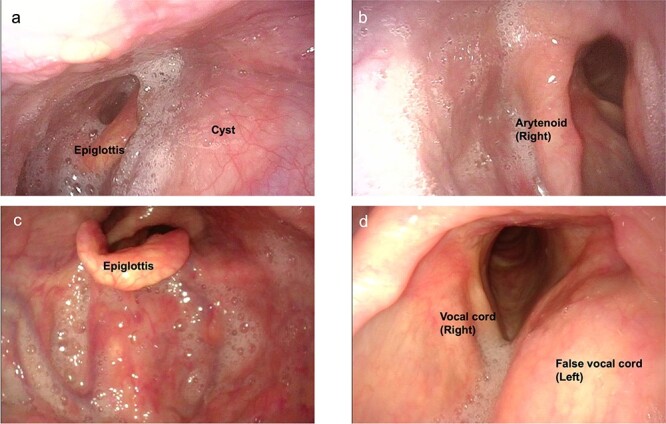
Preoperative endoscopic findings (**a**, **b**) before drainage (**c**, **d**) after drainage.

The neck cyst slowly enlarged, and the patient visited the clinic again in 2021. He had developed mild dysphagia and an oppressive feeling in his throat without pain, dysphonia or hoarseness. Physical examination revealed a huge soft mass on his anterior neck toward the left side, extending from the mandible to the clavicle (Fig. 1a and b). A CT scan exposed a lobulated cystic mass, measuring 18 × 16 cm, extending from the tongue base to the inferior level of the clavicle, forcing the thyroid cartilage toward the right side (Fig. 2a and b). The cyst had dislocated the hyoid bone below the upper level of the thyroid cartilage (Fig. 2c). The thyroid gland showed a normal appearance and position. Flexible laryngoscopy revealed marked swelling of the tongue base and a false vocal cord on the left side, narrowing the airway (Fig. 3a and b).

**Figure 4:**
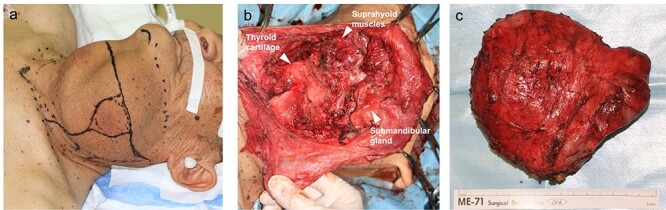
Pre- and intraoperative photographs (**a**) preoperative photographs (**b**) intraoperative photograph (**c**) extracted thyroglossal duct cyst.

**Figure 5:**
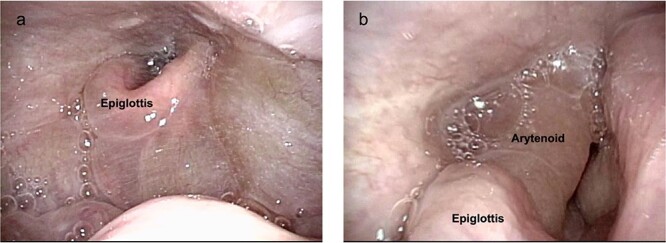
Postoperative endoscopic findings.

Surgical excision of the cyst was planned, and difficult intubation was expected. Three hours before the start of surgery, the cyst was punctured with an 18G needle and most of the fluid was drained (1250 cc). Thereafter, the swelling of the tongue base and false vocal cord was remarkably reduced (Fig. 3c and d). Due to the prior reduction in cyst volume, intubation was performed without complications, and surgery was performed under general anesthesia. Intraoperatively, a decompressed 14 × 13-cm cyst was observed severely deforming the hyoid bone (Fig. 4). The middle section of the bone, which was tightly attached to the cyst, was removed using the Sistrunk procedure. After cyst extraction, elevation of the thyroid cartilage was achieved by suturing it to the suprahyoid muscles. Tracheotomy was performed in anticipation of airway obstruction. Until postoperative day (POD) 7, oral intake was not permitted owing to throat swelling. On POD 8, swallowing videofluorography was performed, and pulmonary aspiration was not observed. The tracheostomy was closed on POD 12 and the patient was discharged on POD 15 without any complications. Histopathological examination confirmed the diagnosis of TGDC, with no evidence of malignancy.

## DISCUSSION

TGDC is one of the most common cystic diseases of the anterior neck. It is generally present in young adults but is rare in older individuals. In our case, the patient showed an atypical clinical course in terms of the age of onset and cyst size. In a previous report on 685 cases, the average size of TGDC was reported to be 2.4 cm (0.4–9.9 cm) [3]. To the best of our knowledge, our patient’s cyst (18 × 16 cm) is the second largest such cyst reported worldwide, following the case of a patient with a cyst 30 × 24 cm in size [6]. As TGDC can be located along the thyroglossal duct pathway, it should be differentiated from other types of neck tumors or cysts that can arise in the anterior neck, such as dermoid cysts, epidermal inclusion cysts, lymphangiomas, ranula, ectopic thyroid and lateral cervical cysts. Consideration of the fact that TGDC could be malignant (incidence: 3.2–7.4%) [3, 7], is important. The major histological type of TGDC-associated carcinoma is papillary thyroid carcinoma, which arises from ectopic thyroid tissue [7].

In our patient, atypical perioperative management was required because of the extreme size of the cyst. We reduced the cyst volume preoperatively to enable safe oral intubation. In previous cases, difficult intubation was also expected, and awake intubation was performed [2, 6]. In the case of large cysts, the possibility of difficult intubation should be mentioned to patients, and preoperative cyst volume reduction should be suggested for the safe induction of general anesthesia. Preoperative volume reduction was effective not only for safe oral intubation but also for intraoperative observation of the normal structure and dislocated hyoid bone. In a previous case, intraoperative decompression by needle aspiration was also performed, and the authors concluded that preoperative aspiration aided in the identification of tissue planes [2]. Although preoperative volume reduction of the cyst is not usually performed in TGDC surgery, it could be an option in the case of a huge cyst.

In terms of postoperative airway management, the necessity of tracheotomy may be controversial. Although tracheotomy has been performed in some cases of TGDC with intralaryngeal extension [8], there were no cases of extralaryngeal TGDC that required tracheotomies, even though these cysts were extraordinarily large [2, 6, 9–11]. Our patient experienced severe edema of the larynx and narrowing of the airway postoperatively (Fig. 5 a and b), suggesting that tracheotomy should be considered in future surgeries depending on cyst size and intraoperative findings.

## CONCLUSION

We present a rare case of a giant TGDC in an older adult patient in which atypical perioperative management was required. In patients with large TGDC, preoperative volume reduction of the cyst and tracheotomy should be considered for oral intubation and postoperative airway management, respectively.
